# 694. Impact of COVID-19 Pandemic on Clostridioides difficile Infection Rates and Risk Factors in Maryland

**DOI:** 10.1093/ofid/ofad500.756

**Published:** 2023-11-27

**Authors:** Kaytlynn Marceaux-Galli, Rebecca Perlmutter, David Blythe, Lucy Wilson

**Affiliations:** Maryland Department of Health, Baltimore, Maryland; Maryland Department of Health, Baltimore, Maryland; Maryland Department of Health, Baltimore, Maryland; University of Maryland Baltimore County, Towson, Maryland

## Abstract

**Background:**

While many Healthcare Associated Infection (HAI) rates increased during the pandemic, inpatient *C. difficile* infection (CDI) rates declined. We compared pre-pandemic (2018-2019) to interpandemic (2020-2021) data, using Maryland’s population-based surveillance data collected through the Emerging Infections Program’s CDI HAIC project, to quantify the impact of COVID-19 on decreases in CDI within different epidemiologic classes (epi classes): HCFO, CO-HCFA, CA (Tables 1 & 2).

**Table 1.**
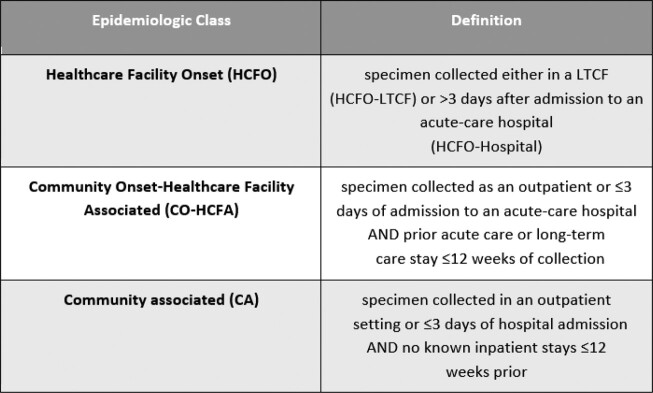
Epidemiologic class definitions for Clostridioides difficile incident (CDI) case classifications determined by the Emerging Infection Program’s HAIC project.

**Table 2.**
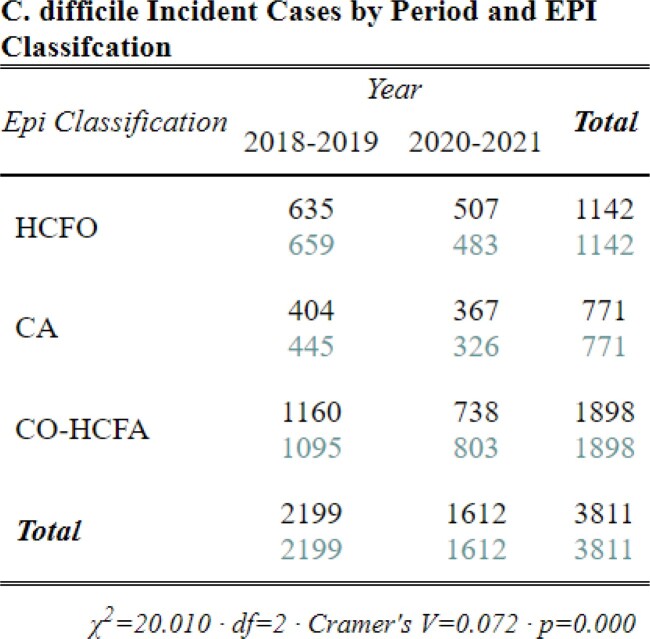
Chi-square crosstabulation of Epidemiologic class Clostridioides difficile incident (CDI) case counts observed and expected pre- and interpandemic. Note: the turquoise-colored text is the expected chi-square case counts while the black text is the observed case counts.

**Methods:**

Using logistic regression, we examined changes in epi class, prior antibiotic use, and basic demographics. We used crosstabulation chi-square analysis to determine proportional differences in case characteristics, pre- and interpandemic. All analyses were conducted using R version 4.2.2.

**Table 3.**
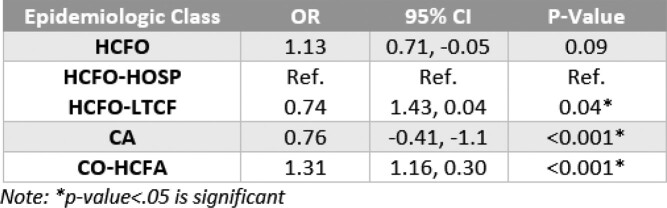
Three independent logistic regression models to determine if epidemiologic case classification is dependent on period of CDI case (Model: Interpandemic = HCFO, Interpandemic = HCFO-LTCF (HCFO-Hospital Ref.), Interpandemic = CA, Interpandemic = CO-HCFA).

**Results:**

Between 2018-2021, we identified 3,822 CDI cases: 1,142 HCFO; 771 CO-HCFA; and 1,898 CA and 11 with incomplete data. Interpandemic cases were 24% less likely to be CA and 31% more likely to be CO-HCFA (Table 3, p< 0.001) than pre-pandemic cases. Though cases were 13% more likely to be classified HCFO, this increase was not significant between periods (Table 3, p=0.09). However, differences were identified between HCFO-LTCF and HCFO-Hospital where HCFO-LTCF cases were 26% less likely interpandemic than HCFO-Hospital cases (Table 4, p< 0.05; Table 3, p=0.04; respectively). Antibiotic use ≤12 weeks prior to collection did not change significantly (Table 5, p=0.7). All-cause hospital admission was 27% less likely interpandemic (p< 0.05). Though our catchment area is predominately White, Black/African American case counts increased while case counts in all other races declined and no differences were observed between sexes (Table 6, p< 0.001; Table 7, p=0.3; respectively).

**Table 4.**
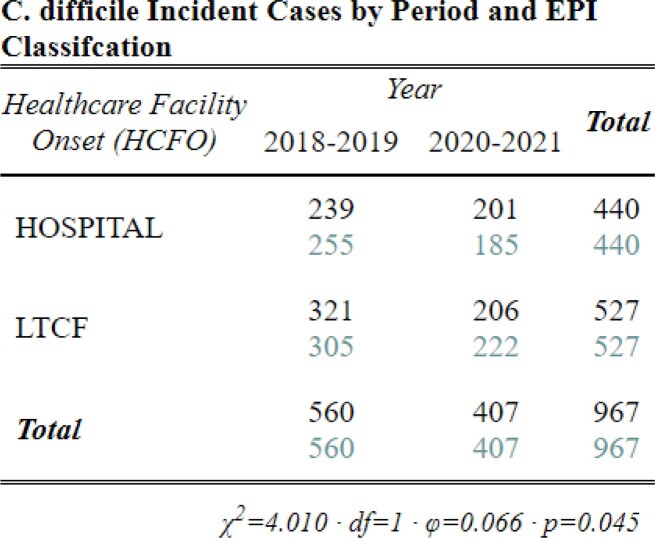
Chi-square crosstabulation of HCFO Epidemiologic class Clostridioides difficile incident (CDI) case counts observed and expected pre- and interpandemic to determine where within HCFO classification between periods declines in CDI were more observed. Note: Not all HCFO designated cases are included ONLY those that were in a LTCF or Hospital 3 days prior to CDI collection date. Also, the turquoise-colored text is the expected chi-square case counts while the black text is the observed case counts.

**Table 5.**
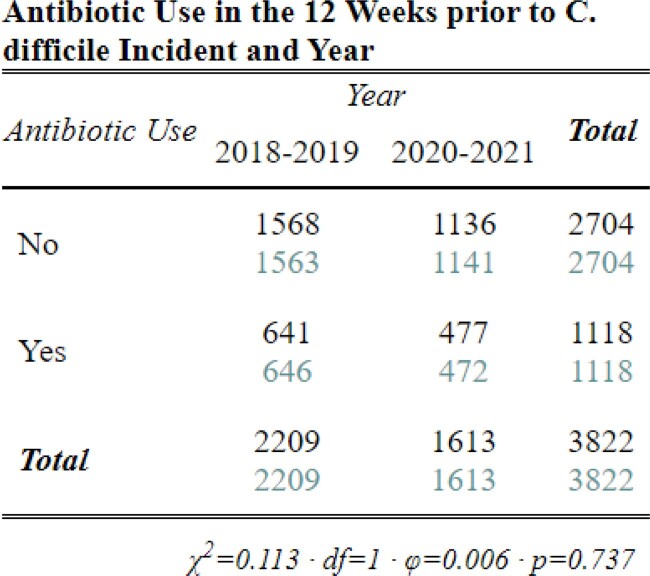
Chi-square crosstabulation comparing CDI case counts with Antibiotic Use 12 weeks prior to C. difficile culture collection date pre-pandemic and interpandemic. Note: the turquoise-colored text is the expected chi-square case counts while the black text is the observed case counts.

**Table 6.**
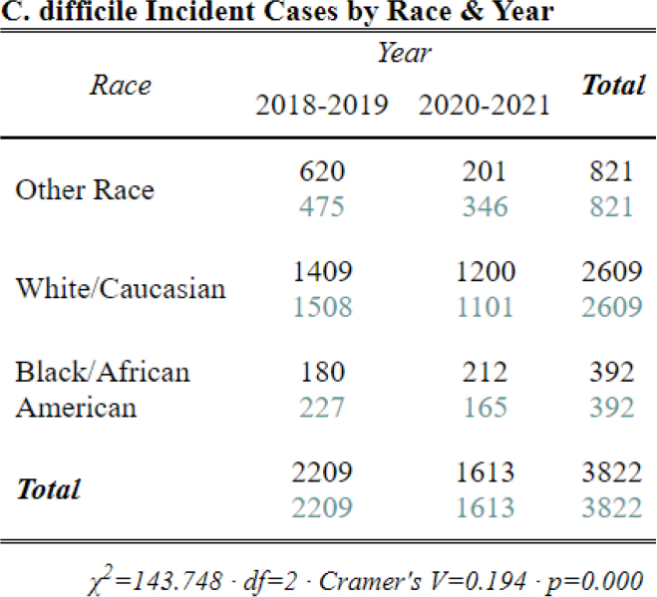
Chi-square crosstabulation comparing CDI case counts of Race pre-pandemic and interpandemic. Maryland’s CDI cases are predominately White/Caucasian with the Black/African American group as the second highest racial group for our surveillance site. Comparing these two groups between these time periods is important to identify the total decline of CDI cases interpandemic. Further investigations should be conducted to better understand why this minority group’s CDI case counts increased during the COVID-19 Pandemic while all others declined. Note: the turquoise-colored text is the expected chi-square case counts while the black text is the observed case counts.

**Conclusion:**

Overall CDI case counts declined during the COVID-19 pandemic across all epi classes. The rate of decline of CA was greater than the decrease in CO-HCFA cases. HCFO-LTCF cases declined more than HCFO-Hospital cases. Unexplained case rate differences between races raise health equity concerns needing exploration. Further investigation may determine whether changes in healthcare seeking behavior, changes in infection prevention methods in LTCFs, local COVID-19 restrictions, or other factors may have impacted CDI rates.

**Table 7.**
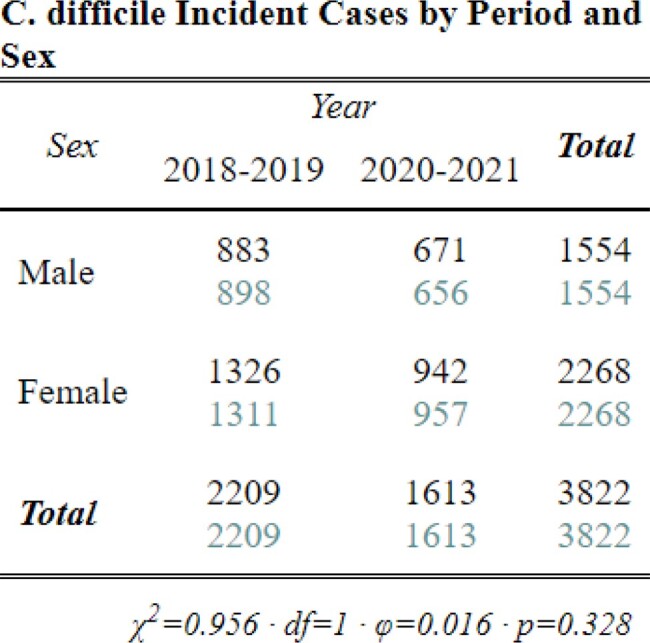
Chi-square crosstabulation comparing CDI case counts of Sex pre-pandemic and interpandemic. Note: the turquoise-colored text is the expected chi-square case counts while the black text is the observed case counts.

**Disclosures:**

**All Authors**: No reported disclosures

